# From Genome-wide Association Studies to Functional Variants: *ARL14* Cis*-*regulatory Variants Are Associated With Severe Malaria

**DOI:** 10.1093/infdis/jiae159

**Published:** 2024-03-26

**Authors:** Mathieu Adjemout, Frederic Gallardo, Magali Torres, Alassane Thiam, Babacar Mbengue, Alioune Dieye, Sandrine Marquet, Pascal Rihet

**Affiliations:** Aix-Marseille Univ, Inserm, TAGC Theories and Approaches of Genomic Complexity, MarMaRa Institute, Marseille, France; Aix-Marseille Univ, Inserm, TAGC Theories and Approaches of Genomic Complexity, MarMaRa Institute, Marseille, France; Aix-Marseille Univ, Inserm, TAGC Theories and Approaches of Genomic Complexity, MarMaRa Institute, Marseille, France; Pole d’Immunophysiopathologie & Maladies Infectieuses, Institut Pasteur de Dakar; Service d’Immunologie, Université Cheikh Anta Diop de Dakar, Senegal; Service d’Immunologie, Université Cheikh Anta Diop de Dakar, Senegal; Aix-Marseille Univ, Inserm, TAGC Theories and Approaches of Genomic Complexity, MarMaRa Institute, Marseille, France; Aix-Marseille Univ, Inserm, TAGC Theories and Approaches of Genomic Complexity, MarMaRa Institute, Marseille, France

**Keywords:** severe malaria, GWAS, *ARL14*, enhancer, promoter

## Abstract

**Background:**

Genome-wide association studies have identified several nonfunctional tag single-nucleotide polymorphisms (SNPs) associated with severe malaria. We hypothesized that causal SNPs could play a significant role in severe malaria by altering promoter or enhancer activity. Here, we sought to identify such regulatory SNPs.

**Methods:**

SNPs in linkage disequilibrium with tagSNPs associated with severe malaria were identified and were further annotated using FUMA. Then, SNPs were prioritized using the integrative weighted scoring method to identify regulatory ones. Gene reporter assays were performed to assess the regulatory effect of a region containing candidates. The association between SNPs and severe malaria was assessed using logistic regression models in a Senegalese cohort.

**Results:**

Among 418 SNPs, the best candidates were rs116525449 and rs79644959, which were in full disequilibrium between them, and located within the *ARL14* promoter. Our gene reporter assay results revealed that the region containing the SNPs exhibited cell-specific promoter or enhancer activity, while the SNPs influenced promoter activity. We detected an association between severe malaria and those 2 SNPs using the overdominance model and we replicated the association of severe malaria with the tagSNP rs116423146.

**Conclusions:**

We suggest that these SNPs regulate *ARL14* expression in immune cells and the presentation of antigens to T lymphocytes, thus influencing severe malaria development.

## Teaser

We identified an *ARL14* cis-regulatory region containing functional single-nucleotide polymorphisms (SNPs) associated with severe malaria and provided evidence of allele-specific promoter activity. Hence, these SNPs could be involved in the development of severe malaria by regulating *ARL14* expression and consequently T-lymphocyte activation.

*Plasmodium falciparum* malaria remains a major health problem in tropical and subtropical countries, causing 619 000 deaths in 2021, mostly in Africa [1]. Subjects infected by *P falciparum* can develop asymptomatic infections, mild malaria, and severe forms of the disease, such as cerebral malaria (CM) or severe anemia. Evidence for host genetic factors controlling the different malaria phenotypes has been reported [2–4]. Furthermore, genome scans have mapped several genes in the control of blood infection levels [5, 6], mild malaria [5, 7], and severe malaria (SM) [8–13]. In particular, the genome-wide association study (GWAS) based on the largest dataset identified different SM loci on chromosomes 1, 2, 3, 4, 6, 9, 10, 11, and 12 [13]. Nowadays, the challenge is to identify the causal variants that explain the association of the tag single-nucleotide polymorphisms (SNPs) with SM. A very small number of potentially causal functional variants have been identified. Hemoglobin S in *HBB* (HbS) is the best-known causal variant. This missense mutation located within chromosome 11 has been shown to be associated with protection against SM [8, 14], to influence parasite growth in vitro in hypoxic conditions [15], and to be associated with low parasitemia in vivo [16]. Besides, the structural variant *DUP4*, which is in linkage disequilibrium (LD) with a tagSNP located within chromosome 4 and associated with SM, was shown to prevent parasite growth in vivo [17]. In addition to these variants affecting the protein sequence, it is thought that regulatory variants explain associations with SM detected by GWAS. More generally, since most of the SNPs associated with a disease are within noncoding regions, it has been proposed that the causal SNPs are mostly SNPs regulating gene expression [18, 19]. In this way, several regulatory variants in LD with the tagSNP located within *ATP2B4* have been proposed as causative variants [20, 21]. Furthermore, several authors have provided evidence of their effect on both the intracellular calcium concentration [20, 21] and the in vitro growth of the parasite [22, 23].

The purpose of this work was to identify new regulatory variants underlying genetic association signals detected by GWAS of SM. To this aim, we first searched for the SNPs in LD with the tagSNPs and prioritized them. Second, we investigated whether the best candidates are regulatory variants. Third, we assessed their association with SM in a Senegalese population.

## MATERIALS AND METHODS

### Bioinformatic Prioritization and Functional Annotation of Genetic Variants

Supplementary Table 1 shows the SNPs whose association with SM has been detected by GWAS [8–13]. Among these, rs334 or HbS that is a causative variant located on chromosome 1 was excluded. SNPs in LD with the 36 associated tagSNPs (Supplementary Table 1) were searched using the FUMA SNP2GENE tool [24]. This tool provided a list of 418 SNPs along with their expression quantitative trait loci (eQTL) annotation and CADD (combined annotation dependent depletion) score, which aims to predict the pathogenicity of genetic variants. SNPs in LD with these tagSNPs, in the African population, were selected with a threshold of *r*^2^ >0.6. They were further prioritized using the integrative weighted (IW) scoring annotation tool [25] and annotated using both ChIP-seq ReMap catalogue and ENCODE database [26, 27]. This helped us identify SNPs located within sequence-binding transcription factors (TFs) or within potential regulatory elements. For the 2 selected SNPs, the impact of the SNPs on TF binding sites (TFBSs) was evaluated with RSAT tool [28], using multiple databases of TFBS (Jaspar, Hocomoco, ENCODE, Homer). To identify the TFs potentially involved in the enhancer activity of *ARL14* promoter, motifs predicted by JASPAR2022 [29] were retrieved using the University of California, Santa Cruz (UCSC) genome browser [30]. Only predicted TFBSs with a score >400 were selected.

### Study Subjects, Blood Samples, and Phenotypes

The study population has been previously described [21, 31]. In brief, it comprised 117 patients with SM and 79 control individuals. Ninety patients with CM and 27 with severe noncerebral malaria (NCM) were enrolled from the principal hospital of Dakar and the regional hospital of Tambacounda. The control samples were taken from healthy individuals living in Dakar. They did not have any malaria infection (as determined by microscopy) or any other febrile illness, selected at the same period of enrollment. The allele and genotype frequencies within the control population reflected those within the general population. Informed consent was obtained from each participant and/or their relatives prior to inclusion, after giving them written or verbal information in their native language. The study received approval from the institutional research ethics committee of Université Cheikh Anta Diop.

SM cases were defined according to the World Health Organization criteria [32]. CM cases were defined based on a deep coma (Glasgow coma score <9), and NCM cases had severe anemia, hypoglycemia, respiratory distress, or hypoxia.

### DNA Extraction, DNA Amplification, and Genotyping

Genomic DNA was extracted and further amplified as described previously [31]. The tagSNP rs116423146 and 2 additional SNPs, namely rs116525449 and rs79644959, were genotyped using the TaqMan allelic discrimination technique (C_150809577_10, C__27835914_10, C__27837814_10; Thermo Fisher, Waltham, Massachusetts). In all, 192 of the 196 individuals were successfully genotyped for the 3 SNPs. The reaction mixture consisted of 1 µL of genomic DNA at 12.5 ng/µL, 2.1 µL of 2× master mix (TaqMan Genotyping Master Mix, Applied Biosystems, Waltham, Massachusetts), and 0.06 µL for the TaqMan 40× assays in a final volume of 5 µL.

### Luciferase Gene Reporter Assay

#### Promoter Activity

The 460 bp promoter region located upstream of the *ARL14* gene (GRCh38, chr3: 160676881-160677341 containing the SNPs rs79644959, rs116525449) was cloned into the pLG.4.12-basic plasmid (catalog # E6671, Promega, Madison, Wisconsin) at the HindIII and BglII restriction sites (GeneCust, Boynes, France). We obtained 2 different pGL4.12 constructs containing the minor or major alleles of both SNPs. The reporter gene experiment was performed in K562 (CCL-243, ATCC) and GM12878 (Coriell Institute) cell lines. K562 cells were grown in RPMI 1640 (Thermo Fisher) with glutaMax (61870-044, Gibco) and 10% decomplemented fetal bovine serum (FBS). The GM12878 cells were cultured in RPMI 1640 with glutaMax (61870-044, Gibco) and 15% nondecomplemented FBS. Transfection of cells was performed using the Neon transfection system (Invitrogen, Thermo Fisher) according to the manufacturer's instructions. For each assay, 10^6^ cells were co-transfected with 200 ng of control vector pRL-SV40 (catalog # E2231, Promega) and 1 µg of vector tested. Empty pGL4.12-basic vector was used as a control. After transfection, cells were maintained at 37°C in 5% carbon dioxide for 24 hours. Luc2CP and Renilla luciferase values were obtained by analyzing 20 μL of cell lysate.

#### Enhancer Activity

The 460 bp *ARL14* promoter region used previously was cloned into pGL3-SV40 vector (catalog # E1761, Promega) between BamH1 sites. pGL3-SV40 vector was used as a control. Luciferase assays were performed in K562 and GM12878 cells as described above.

### Reverse-Transcription Quantitative Polymerase Chain Reaction

The RNA extraction from both K562 cells and GM12878 cells was conducted using the RNeasy Plus mini kit (Qiagen, Hilden, Germany). Following this, 1 µg of RNA per sample was converted into cDNA using the Superscript VILO Master Mix (Invitrogen, Thermo Fisher Scientific). Real-time quantitative PCR (RT-qPCR) was carried out using the SYBR Select Master Mix (Thermo Fisher Scientific). Primers were designed employing the Primer 3 software. Subsequently, gene expression was normalized against glyceraldehyde 3-phosphate dehydrogenase, and the relative expression was computed using the ΔCt method. The data provided is an average from triplicates across 3 independent experiments per sample and is expressed as fold change relative to K562 cells.

### Statistical Analysis

The Haploview tool [33] was used to determine the deviation in genotype frequency from Hardy-Weinberg equilibrium for the control group. Genetic association analyses were performed using IBM SPSS Statistics 27 (IBM, Armonk, New York). We performed logistic regression analyses that allowed us to include age, sex, and HbS in the model. For luciferase reporter assay and RT-qPCR results, statistical analyses were performed using the Wilcoxon test. All the tests used were 2-sided. *P* values <.05 were considered statistically significant. Plot generation was performed using ggplot2.

## RESULTS

### Prioritization of Candidate SNPs Identified 2 SNPs Close to *ARL14*

**Figure 1.**
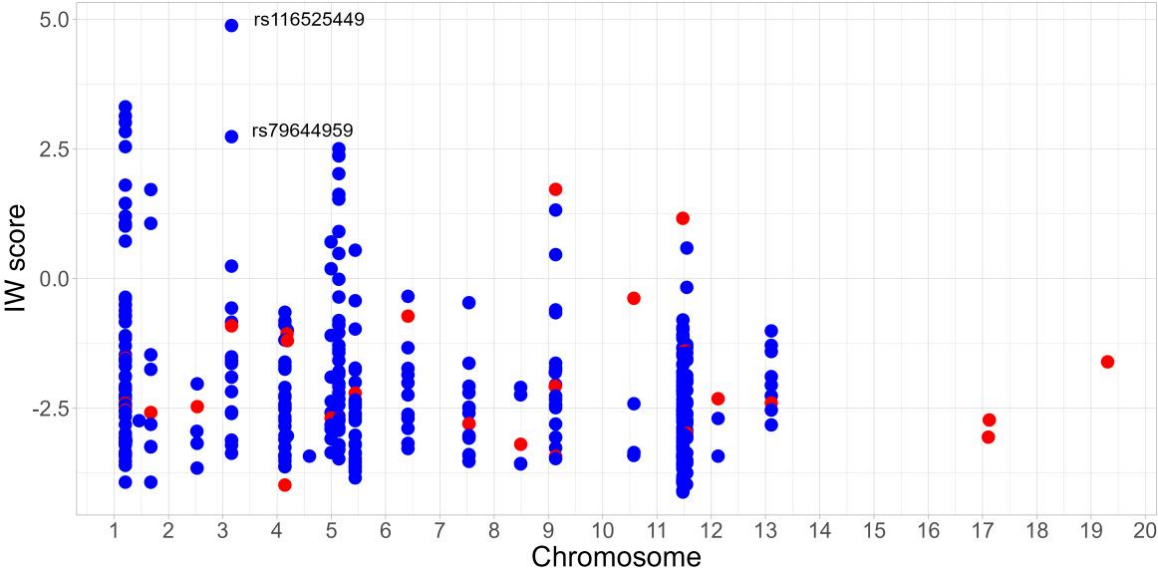
Manhattan plot showing candidate single-nucleotide polymorphism (SNP) integrative weighted (IW) scores. The IW scoring method was used to prioritize functionally relevant noncoding variants. The graph displays the IW scores of 418 candidate SNPs depending on their position. The 36 tagSNPs, mostly low-ranked, are shown in red dot. The SNPs in linkage disequilibrium with the tagSNPs are shown in blue dot. The best candidate was rs116525449.

We sought to identify functional regulatory SNPs underlying the genetic association signals detected by GWAS. Among the various GWAS, a total of 36 tagSNPs associated with SM were found to be distributed across 29 loci (Supplementary Table 1). Then, we identified the SNPs in LD with the malaria-associated tagSNPs in African population, using an *r*^2^ threshold of 0.6. Finally, we prioritized 418 SNPs using the IW scoring method. Findings are summed up in Figure 1, and the ranking of all SNPs is shown in Supplementary Table 2. Table 1 presents the SNPs with the 10 highest IW scores. They were clustered into 3 loci: *ARL14*, *ATP2B4*, and *KLHL3*. The SNP having the best score is located on chromosome 3 near *ARL14* (Figure 1). Interestingly, rs116525449 is located at 31 bp of rs79644959, which was ranked 6th based on IW score. Furthermore, these 2 SNPs are in full LD in African populations based on 1000 genome data. Both SNPs are located within promoter activity region based on an ENCODE annotation (EH38E2253584). The other SNPs were also within regulatory regions, as shown in Table 1. Eight of the top 10 SNPs were eQTLs for *ATP2B4*, *LAX1*, or *KLHL3*, while rs116525449 and rs79644959 were not annotated as eQTLs. Furthermore, we investigated the ability of the sequences containing the SNPs to bind TFs using the ReMap tool, which is a ChIP-seq peak catalogue. The best SNPs shown in Table 1 were all located in peaks of ChIP-seq. These include rs116525449 and rs79644959, for which 86 and 103 peaks of ChIP-seq were registered in the ReMap catalogue, respectively. Interestingly, the SNP with the highest IW score is close to *ARL14*, which has been poorly studied in malaria. Also, we further focused on rs116525449 and rs79644959 located close to *ARL14*. We hypothesized that they regulate *ARL14* expression, leading to malaria resistance or susceptibility.

**Table 1.** Numbers of Transcription Factor ChIP-seq Peaks, Expression Quantitative Trait Loci Hit, and ENCODE Candidate Cis-regulatory Elements for the Best Single-Nucleotide Polymorphism Prioritized by Integrative Weighted Scoring Method

Chr	SNP rsID	Rank IW Scoring	Binding Transcription Factor	eQTL Hits	Regulatory Element
3	rs116525449	1	86	…	Promoter-like (ARL14)
1	rs11240734	2	76	ATP2B4 (+1)	Promoter-like (ATP2B4)
1	rs1541252	3	168	ATP2B4 (+1)	Promoter-like (ATP2B4)
1	rs10751450	4	79	ATP2B4 (+1)	Proximal enhancer-like
1	rs2228445	5	23	ATP2B4 (+1)	Distal enhancer-like
3	rs79644959	6	103	…	Promoter-like (ARL14)
1	rs1541254	7	214	LAX1	Promoter-like (ATP2B4)
5	rs73298155	8	277	KLHL3 (+7)	Proximal enhancer-like
5	rs11386045	9	4	KLHL3 (+7)	Distal enhancer-like
5	rs2905584	10	20	KLHL3 (+8)	…

Abbreviations: Chr, chromosome; eQTL, expression quantitative trait loci; IW, integrative weighted; SNP, single-nucleotide polymorphism.

### The Sequence Containing rs116525449 and rs79644959 Has a Regulatory Activity in GM12878 and K562 Cell Lines

**Figure 2.**
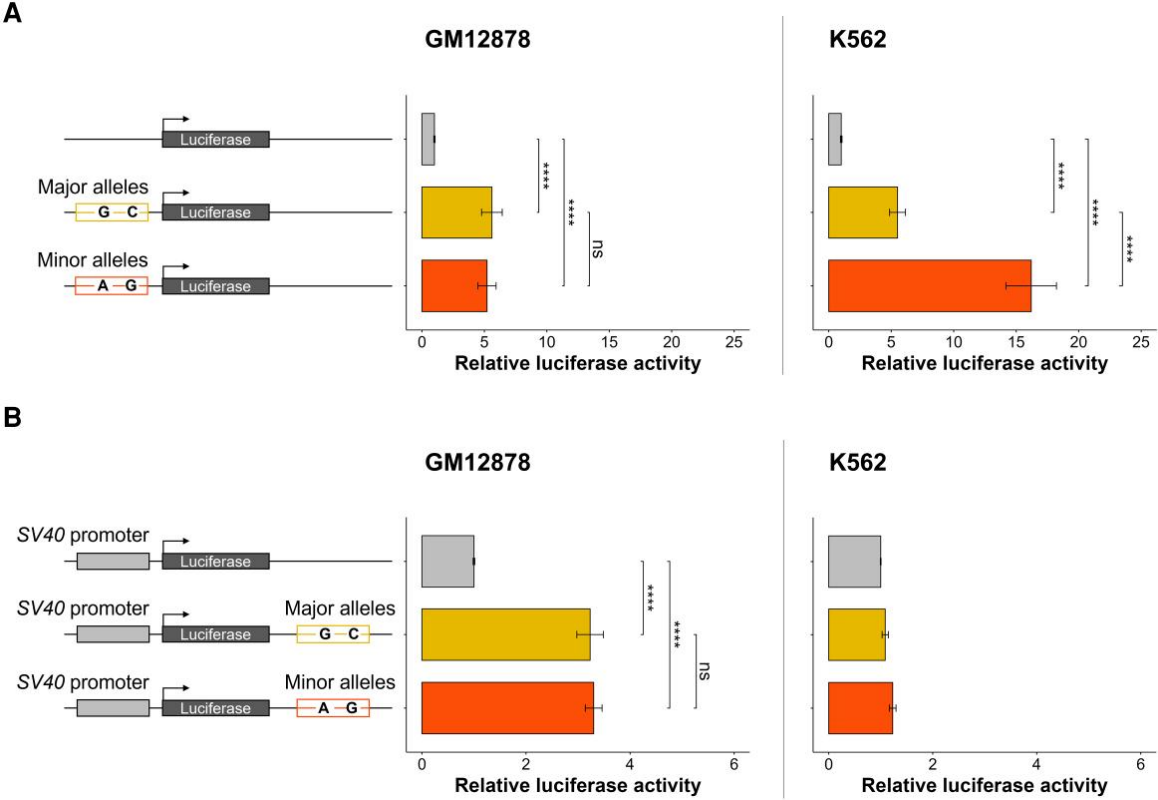
Luciferase gene reporter assays assessing the regulatory activity of *ARL14* variants in GM12878 cells and K562 cells. *A*, Promoter activity of the *ARL14* region containing major or minor alleles of the rs79644959 and rs116525449. Basic vector was used as reference. *B*, Enhancer activity of the *ARL14* region containing major or minor alleles of rs79644959 and rs116525449. SV40 promoter was used as reference. Data presented as the mean ± standard error of the mean of 9 replicates. ****P < .0001; ns, nonsignificant.

We investigated the potential regulatory effect of rs116525449 and rs79644959 using luciferase reporter assays in GM12878 and K562 cell lines, which are lymphoblastoid and erythromyeloid cells, respectively. Since those SNPs are in complete LD, we studied the effect of sequences containing rs79644959-G and rs116525449-C on the one hand and rs79644959-A and rs116525449-G on the other hand, which are the major and minor haplotypes, respectively. First, we cloned the sequences upstream of the luciferase reporter gene to evaluate their promoter activity (Figure 2*A*). Both sequences showed higher luciferase activity than the negative control in GM12878 and K562 cells (*P* < .0001). A 5-fold increase of luciferase activity was observed for both major and minor haplotypes in GM12878 cells and for the major haplotype in K562 cells compared to the activity of the basic vector. Moreover, in K562 cells, the promoter activity was nearly 3-fold higher with the sequence containing the minor haplotype compared with the major haplotype. These results indicate an allele-independent promoter activity in GM12878 cells, while the promoter activity depends on SNP alleles in K562 cells.

We hypothesized that our sequences act as enhancers on distal genes. The results of FUMA showed evidence of interaction between the region containing SNPs and *NMD3* and *PP1ML* promoters in GM12878. Hence, we cloned our sequences downstream of the luciferase reporter gene. Strikingly, our sequences showed an enhancer activity in GM12878 cells (*P* < .0001), but not in K562 cells (Figure 2*B*). The luciferase activity was 3-fold higher in the presence of our sequences in GM12878 cells. However, the enhancer activities of the 2 sequences corresponding to the 2 haplotypes did not differ.

**Figure 3.**
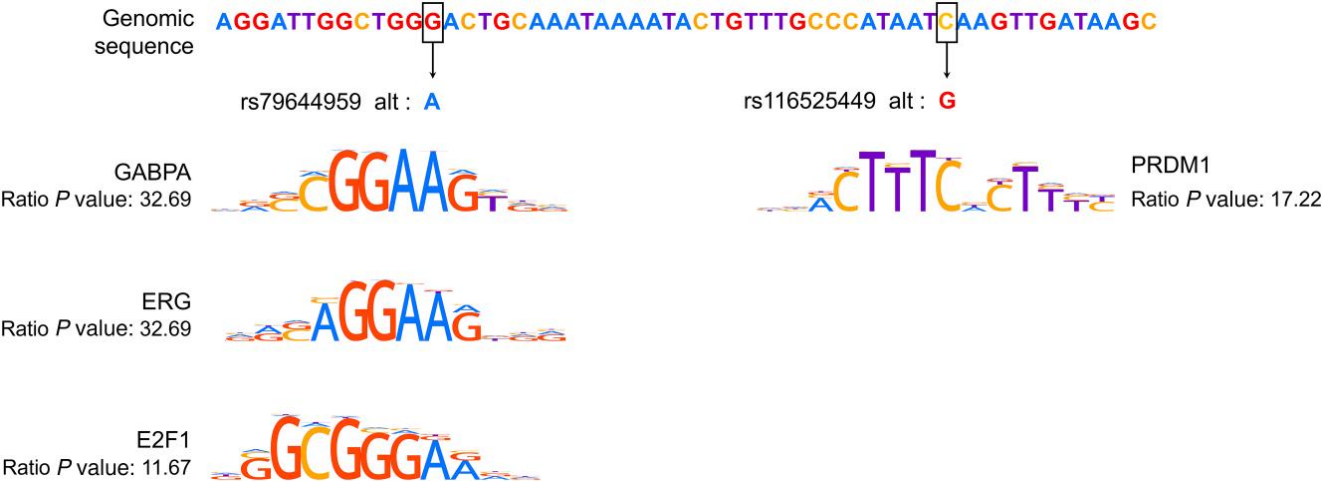
Predicted disrupted transcription factor binding sites by *ARL14* variants using RSAT. Genomic region containing rs79644959 and rs116525449 (hg38-chr3:160677111-160677166) is shown. *P* value ratio was calculated by dividing the best probability of transcription factor binding by the worst probability. GABPA, ERG, and E2F1 had a higher binding probability on rs79644959 minor allele compared to the major allele. PRDM1 had a higher binding probability on rs116525449 major allele compared to minor allele.

**Figure 4.**
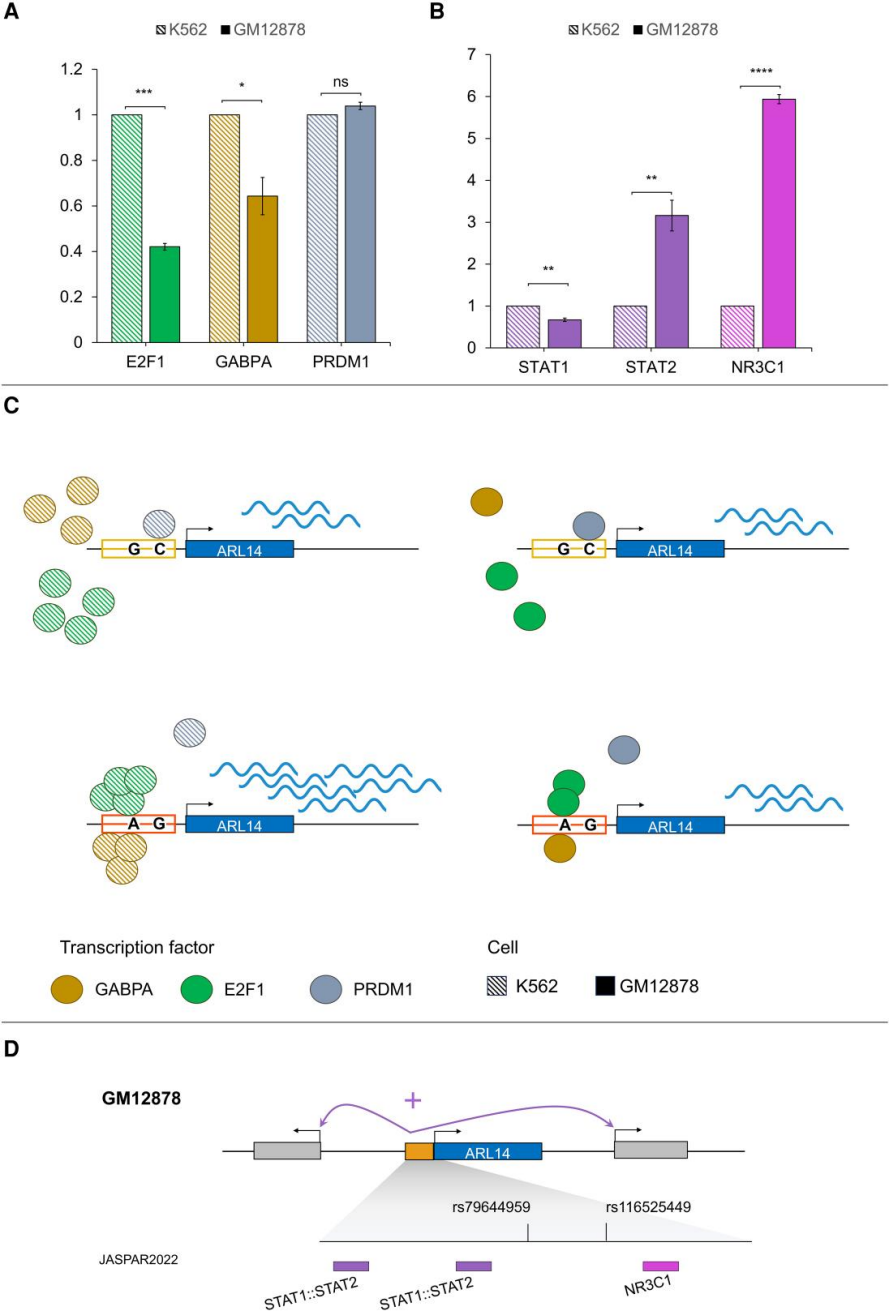
Real-time quantitative polymerase chain reaction (RT-qPCR) results and models for transcriptional regulation of *ARL14* gene in GM12878 and K562 cells. *A* and *B*, RT-qPCR results for selected transcription factor expression levels in GM12878 and K562 cells, respectively. *C*, Model for a promoter activity of the sequence containing rs79644959 and rs116525449. E2F1 and GABPA are expressed in GM12878 and K562 and were predicted to bind to rs79644959 minor allele. PRDM1, which is expressed in GM12878, was predicted to bind to the rs116525449 major allele. As a result, the level of *ARL14* expression in K562 is similar to that in GM12878. *D*, Model for an enhancer activity of the sequence containing rs79644959 and rs116525449. STAT1:STAT2 and NR3C1 that bind to sequences close to the single-nucleotide polymorphisms are affected by neither rs79644959 nor rs116525449. A higher expression of *STAT2* and *NR3C1* in GM12878 causes an increased enhancer activity compared to K562. *P < .05, **P < .01, ***P < .001, ****P < .0001; ns, nonsignificant.

To decipher the molecular mechanisms underlying cell and allele-specific promoter activity, we then investigated whether the 2 SNPs could disrupt TFBS binding to DNA. First, we identified the TFs, for which there is experimental evidence of their binding to the region containing the SNPs based on the ReMap catalogue (Supplementary Table 3). Second, we used RSAT tools to identify TFs, the binding sites of which may be altered by the 2 SNPs (Supplementary Table 4). As shown in Figure 3, the results indicate that rs79644959 influences the binding of GABPA, ERG, and E2F1, while rs116525449 may alter the binding of PRDM1. Besides, based on data of RNA-seq ENCODE cell lines [34], *GABPA*, *E2F1*, and *PRDM1* were shown to be expressed in both K562 and GM12878, while *ERG* was undetected in both cell lines. We further measured *ERG*, *GABPA*, *E2F1*, and *PRDM1* expression levels in GM12878 and in K562 cells using the RT-qPCR method. There was no ERG expression in GM12878 or K562. In addition, we found that *E2F1* and *GABPA* levels were 2-fold and 1.3-fold higher in K562 cells than in GM12878, respectively, while we found no difference between *PDRM1* expression level in K562 and GM12878 cells (Figure 4*A*). We propose that the combination of high of *E2F1* and *GABPA* expression levels, along with their preferential binding to the minor allele of rs116525449, results in a higher promoter activity of the minor allele sequence compared to the major allele sequence in K562 (Figure 4*C*). Furthermore, the PRDM1 binding to the rs116525449 major allele may compensate for the low *E2F1* and *GABPA* expression in GM12878, possibly explaining the lack of SNPs impact on promoter activity in GM12878.

We then investigated whether the binding of certain TFs to our sequences could explain the allele-independent but cell-specific enhancer activity. Sequence analysis using JASPAR allowed us to predict TFBS (Supplementary Table 5), including *STAT1*, *STAT2*, and *NR3C1* binding sites. Interestingly, their expression levels were found to be the highest in GM12878 cells of all predicted TFs based on data of RNA-seq ENCODE cell lines [34]. Using RT-qPCR, we found that *NR3C1* and *STAT2* levels were 6.1-fold and 3.6-fold higher in GM12878 than in K562, respectively (Figure 4*B*). *STAT1* levels were, nevertheless, 1.5-fold lower in GM12878 than in K562. Interestingly, TFBS do not align to the SNPs, indicating that *STAT1*, *STAT2*, and *NR3C1* bind to sequences near the SNPs (Figure 4*D*). These results indicate an allele-independent enhancer activity and an increased enhancer activity in GM12878 compared to K562.

### rs116525449 and rs79644959 Variants and Their GWAS tagSNP Are Associated With SM in Senegal

We further investigated the association of *ARL14* SNPs with SM. To this aim, we selected rs116423146, which was previously identified by a GWAS [13], and rs116525449 and rs79644959 for genotyping in a Senegalese population. There was no deviation from the Hardy-Weinberg equilibrium (*P* = 1), and there was no difference between the frequency of genotypes in the control group and that of the general population living in The Gambia, based on the Gambian Genome Variation Project (*P* > .39). Furthermore, we evidenced full LD between rs116525449 and rs79644959 as predicted by the 1000 Genome data. However, the LD coefficient between rs11623146 and the 2 SNPs was lower in our Senegalese cohort (*r*^2^ = 0.47) than that in the whole 1000 Genome African population (*r*^2^ = 0.65) and that in the Niger population (*r*^2^ = 0.83). It was, however, higher than that of the Gambian population (*r*^2^ = 0.33). Nonetheless, we replicated the association of the tagSNP rs11623146 with SM.

The *P* value was significant (*P* = .030) using the overdominance model (Table 2), which is the most appropriate inheritance model, as stated in the previous study [13]. Despite the low LD between the tagSNP rs11623146 and regulatory SNPs rs116525449 and rs79644959, we also found a significant association (*P* = .017). All *P* values remained significant (*P* < .05) when including age, which was the only covariate with a significant effect (Table 2). It is interesting to note that the *P* value was improved for both rs116525449 and rs79644959 compared to the tagSNP. We further included age, the tagSNP, and either rs116525449 or rs79644959 in the regression logistic model to determine whether the association of the tagSNP rs116423146 could be explained by the effect of the other SNPs. After adding either rs116525449 or rs79644959 to the statistical model, the association of rs11623146 with SM was no longer significant. Conversely, both rs116525449 and rs79644959 remained associated with SM. Our results suggest that SNPs rs116525449 and rs79644959 could be the causal SNPs.

**Table 2.** Association of *ARL14* Single-Nucleotide Polymorphisms With Severe Malaria in a Senegalese Population

SNP rsID	Position^a^	Minor Allele	Risk Genotype	Odds Ratio	95% Confidence Interval	*P* Value
rs116423146 (C > T)	160 679 075	T	CC-TT	2.62^b^ (2.70)^c^ (1.90)^d^	1.07–6.41^b^ (1.04–6.99)^c^ (.63–5.68)^d^	.030^b^ (.041)^c^ (.250)^d^
rs79644959 (G > A)	160 677 123	A	GG-AA	2.29^b^ (2.20)^c,d^	1.15–4.57^b^ (1.07–4.52)^c,d^	.017^b^ (.032)^c^ (.031)^d^
rs116525449 (C > G)	160 677 154	G	CC-GG	2.29^b^ (2.20)^c,d^	1.15–4.57^b^ (1.07–4.52)^c,d^	.017^b^ (.032)^c^ (.031)^d^

^a^Data represent the position on chromosome 3 according to human hg38 coordinates.

^b^Results of the logistic regression analysis for each single-nucleotide polymorphism (SNP) without any covariate.

^c^Results of the logistic regression analysis for each SNP when considering age as a covariate.

^d^Results of the logistic regression model including either rs79644959 or rs116525449, and rs116423146 and age.

## DISCUSSION

In this study, we prioritized 418 candidate SNPs, the top 10 of which were in or near *ARL14*, *ATP2B4*, or *KLHL3.* The best new promising candidate, namely rs116525449, is close to *ARL14* and is in full LD with rs79644959, which was ranked sixth based on the IW score. We further obtained evidence of the functional effect of *ARL14* SNPs and their association with SM.

Our results concerning *ATP2B4* SNPs are reminiscent of those we recently published. We prioritized the SNPs in LD with tagSNPs, whose association had been replicated for SM [21]. The best candidate SNPs were located within *ATP2B4* that encodes for PMCA4, the main calcium pump in red blood cells. Among these SNPs, we reported rs11240734, rs1541252, rs107511450, and 1541254, which were ranked in the top 10 SNPs in the present study. Our prioritization analysis also revealed SNPs located near or in *KLHL3* or *ARL14*, which have been reported to be associated with SM, and the association of which has not been replicated to our knowledge [12, 13].

Here we confirmed for the first time the association between SM and rs116423146, which is close to *ARL14*, using the overdominance model. This result is consistent with the association of SM with rs75731597, which is in LD with rs116423146 [35]. Interestingly, the overdominance model has appeared to be the best genetic model for detecting the association of SM with rs116423146 and rs75731597 [13, 35]. More generally, it has been recently proposed that heterozygote advantage may be detected for other loci, and that overdominance should be considered in GWAS [36]. Since rs116423146 is unlikely functional based on our prioritization and annotation studies, we further investigated the role of the best candidates close to *ARL14*. Here we provide evidence of an association of SM with rs116525449 and rs79644959, which were in LD with rs116423146 in our study population. Interestingly, the LD coefficient varies greatly between African populations, and the LD coefficient in our study population appeared to be intermediate compared with other African populations—it ranges from 0.33 in The Gambia to 0.83 in Nigeria. Strikingly, rs116525449 and rs79644959 seemed to be better associated with SM compared to rs116423146. In the same way, the results based on a conditional logistic regression approach showed that the association between SM and the tagSNP rs116423146 was explained by rs116525449 or rs79644959, suggesting their causal roles.

Furthermore, we provide experimental evidence that rs116525449 and rs79644959 are regulatory SNPs. These variants modify *ARL14* promoter activity in K562 cells, an erythro-myeloid cell line, although those SNPs are not annotated as eQTLs current databases. This is probably due to the absence of rs116525449 and rs79644959 in European populations, which have been in the majority in eQTL studies.

However, we did not detect any effect of the 2 SNPs on gene expression in GM12878 cells, which are immortalized B cells. This suggests a cell-specific regulatory role of rs116525449 and rs79644959. Analyzing TFBS and assessing predicted TF expression levels allowed us to propose a biological model at the molecular level (Figures 4*C* and 4*D*). E2F1 and GABPA may bind to the minor allele of rs79644959 with a higher affinity compared to that of the major allele both in GM12878 and K562 cells. However, the transcription factor PRDM1 that is expressed in GM12878 cells may bind to the major allele of rs116525449, thus compensating for the absence of E2F1 and GABPA binding on the major allele and balancing the expression of *ARL14*.

Moreover, luciferase reporter assay results showed that the sequence containing rs116525449 and rs79644959 has an enhancer activity, which was much higher in GM12878 than that in K562, irrespective of allele. This suggests an allele-independent cell-specific effect. This may be due to differences in TF expression level between GM12878 and K562. In particular, STAT2 and NR3C1 that bind to sequences close to rs116525449 and rs79644959 show higher expression levels in GM12878 cells than those in K562 cells. Furthermore, these TFs are known to be recruited to enhancer regions [37, 38], which would likely activate enhancer activity in GM12878 cells to a greater extent than of K562 cells. *ARL14* genetic variation was previously found to be associated with immune disorders such as systemic sclerosis, systemic lupus erythematosus, rheumatoid arthritis, and idiopathic inflammatory myopathies [39]. Indeed, the small GTPase encoded by *ARL14* plays a key role in the transport of major histocompatibility complex (MHC) class II antigen in dendritic cells, which are professional myeloid antigen-presenting cells [40]. We therefore propose that the regulatory variants rs116525449 and rs79644959 of *ARL14* may influence immune-mediated resistance to SM by influencing the distribution of MHC class II antigens on the surface of antigen-presenting cells and hence activation of T lymphocytes. Increased antigen presentation by such cells could thus enable parasite elimination; however, hyperactivation of CD8 T lymphocytes could be deleterious [41].

In conclusion, our study confirms the association between SM and the tagSNP rs116423146 previously detected by GWAS. Furthermore, we showed that 2 SNPs in LD with rs116423146 are regulatory variants and are associated with SM, suggesting that they are causal variants. Additional genetic association studies in independent African populations would be useful, while further studies are required to investigate their functional effect at the cellular level.

## Supplementary Data

Supplementary materials are available at *The Journal of Infectious Diseases* online (http://jid.oxfordjournals.org/). Supplementary materials consist of data provided by the author that are published to benefit the reader. The posted materials are not copyedited. The contents of all supplementary data are the sole responsibility of the authors. Questions or messages regarding errors should be addressed to the author.

## Supplementary Material

jiae159_Supplementary_Data

## Notes

***Acknowledgments.*** We thank all the patients who participated in the study.

***Financial support*.** This work was supported by the African Higher Education Centers of Excellence project (CEA-SAMEF) at Université Cheikh Anta Diop de Dakar (to A. D.); the Pasteur Institutes in Dakar and in Paris (to A. T.); Inserm (to P. R.); and Aix-Marseille University (to P. R.). M. A. was supported by a PhD fellowship from the French Ministry of Research. This project was carried out in the framework of the Inserm GOLD Cross-Cutting program (to P. R.).
